# Sodium-glucose co-transporter 2 inhibitors: a pleiotropic drug in humans with promising results in cats

**DOI:** 10.3389/fvets.2025.1480977

**Published:** 2025-02-28

**Authors:** Aline B. Vieira, Sarah M. Cavanaugh, Bianca T. Ciambarella, Marcus V. Machado

**Affiliations:** ^1^Biomedical Sciences Department, College of Veterinary Medicine, Cornell University, Ithaca, NY, United States; ^2^Department of Clinical Sciences, Ross University School of Veterinary Medicine, Basseterre, Saint Kitts and Nevis; ^3^Laboratory of Ultrastructure and Tissue Biology, Anatomy Department, Biology Institute, State University of Rio de Janeiro, Rio de Janeiro, Brazil; ^4^Department of Biomedical Sciences, Ross University School of Veterinary Medicine, Basseterre, Saint Kitts and Nevis

**Keywords:** feline diabetes mellitus, type-2 diabetes mellitus, bexagliflozin, velagliflozin, kidney disease, heart disease, cognitive dysfunction, glucose transporters

## Abstract

Diabetes mellitus is a common metabolic disease in humans and cats. Cats share several features of human type-2 diabetes and can be considered an animal model for this disease. In the last decade, sodium-glucose transporter 2 inhibitors (SGLT2i) have been used successfully as a class of hypoglycemic drug that inhibits the reabsorption of glucose from the renal proximal tubules, consequently managing hyperglycemia through glycosuria. Furthermore, SGLT2i have been shown to have cardiac, renal, and other protective effects in diabetic humans acting as a pleiotropic drug. Currently, at least six SGLT2i are approved by the Food and Drug Administration (FDA) for use in humans with type-2 diabetes, and recently, two drugs were approved for use in diabetic cats. This narrative review focuses on the use of SGLT2i to treat diabetes mellitus in humans and cats. We summarize the human data that support the use of SGLT2i in controlling type-2 diabetes and protecting against cardiovascular and renal damage. We also review the available literature regarding other benefits of these drugs in humans as well as the effects of SGLT2i in cats. Adverse effects related to the use of these hypoglycemic drugs are also discussed.

## 1 Introduction

**Figure 1:**
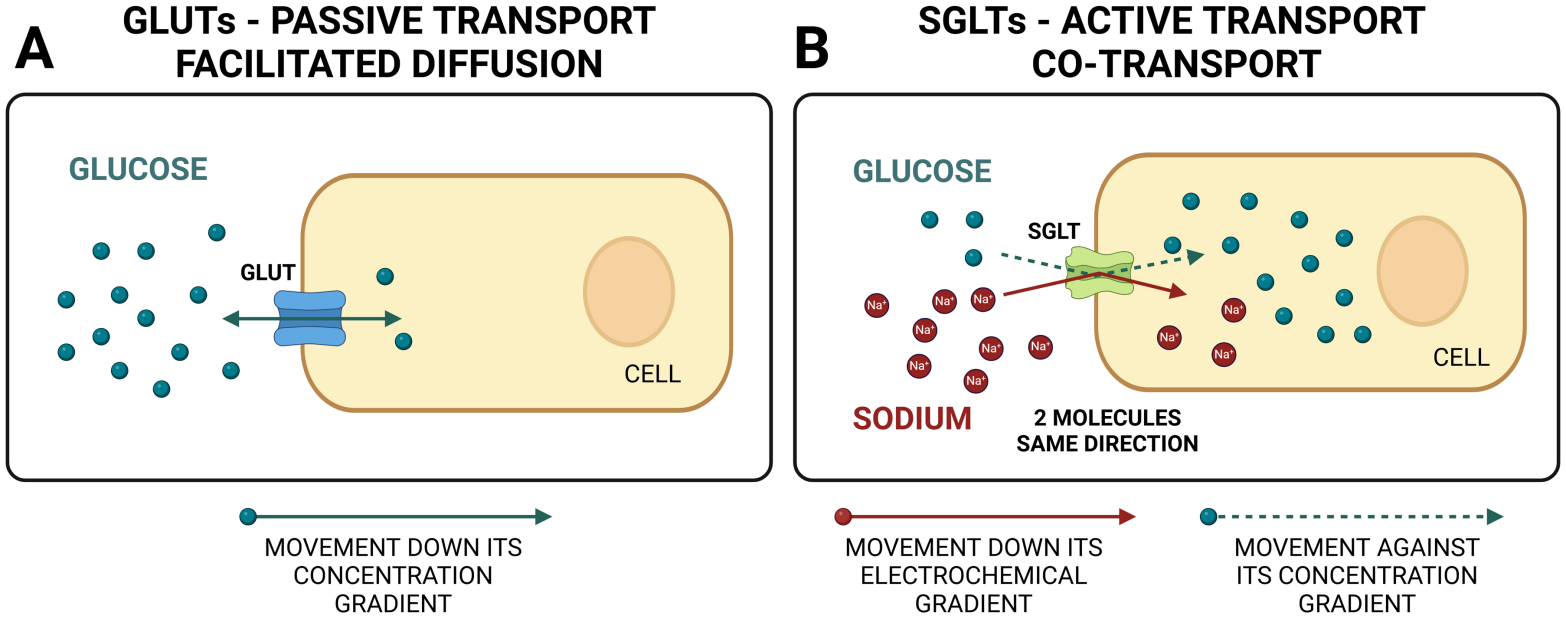
Comparison between GLUTs (glucose transporter facilitators) and SGLTs (sodium-glucose co-transporters). GLUTs use facilitated diffusion (passive transport) for bidirectional glucose transport. SGLTs use active transport to move two molecules in the same direction, one down and one against concentration gradient (co-transport). Created in BioRender. Ciambarella, B. (2025) https://BioRender.com/v21n984.

Glucose is an important energy source for cells and a substrate for many biochemical reactions. As a lipophobic substance, glucose cannot cross the membrane lipid bilayer to enter the cells throughout the body. Integral proteins located in the membrane of every cell called glucose transporters are responsible for glucose transport across the membrane. Glucose can be transported into the cell using facilitated diffusion (passive transport of substances across a biological membrane from an area of higher concentration to an area of lower concentration with the help of a carrier protein) or using co-transport (active transport of two molecules in the same direction with the help of a transport protein called symporter). Symporters rely on the ion moving down the electrochemical gradient (e.g., sodium) to allow the other molecule to move against the concentration gradient (e.g., glucose) (Figures 1A,B).

There are two main types of glucose transporters in the body. The first type is called GLUT (glucose transporter), and they use facilitated diffusion. GLUT proteins are encoded by the SLC2 genes and are members of the major facilitator superfamily of membrane transporters (Figure 1A). They are numbered according to their order of discovery. Currently, 14 GLUT proteins are expressed in humans and categorized into three classes based on sequence similarity: Class I (GLUTs 1–4, 14), Class II (GLUTs 5, 7, 9, and 11), and Class III (GLUTs 6,8,10,12 and 13 [H+ myo-inositol transporter or HMIT]). The second type is called SGLT (sodium-glucose linked transporter or sodium-glucose co-transporter) and relies on co-transport (1) (Figure 1B). SGLTs belong to the mammalian solute carrier family SLC5. This family includes 12 different members in humans that mediate the transport of sugars, vitamins, amino acids, or smaller organic ions such as choline. The SLC5 family belongs to the sodium symporter family (SSS), which encompasses transporters from all kingdoms of life. A summary of the main types of SGLTs present in the human body is presented in Table 1. Two SGLTs (1 and 2) are the most important ones affecting glucose transport in the kidneys (2–5).

**Table 1:** Types, location, function, and characteristics of major sodium-glucose transporters (SGLT).

SGLT types	Location	Function	Characteristic
SGLT 1	The apical membrane of small intestinal cells	Glucose absorption from the intestines	High affinity for glucose, low capacity
	Distal (S3) cells of the proximal tubule	Glucose reabsorption from urine filtrate (10%)	High affinity for glucose, low capacity
SGLT 2	Proximal convoluted tubule (S1 and S2) cells	Glucose reabsorption from urine filtrate (90%)	High affinity for glucose, high capacity
SGLT 3	Intestine, testes, uterus, lungs, brain, thyroid gland	Glucose sensor for controlling glucose levels in the gut and brain. High affinity for iminosugars (carbohydrate analogs)	Low affinity for glucose, high capacity
SGLT 4	Intestine, kidney, liver, brain, lung, uterus, pancreas	High affinity for absorption or reabsorption of mannose. Less affinity for 1,5-anhydro D-glucitol, fructose, and glucose	Lower affinity for glucose compared to mannose, capacity unknown
SGLT 5	Renal cortex	Transport of mannose and fructose with high affinity and galactose, glucose, and 1,5 anhydro D-glucitol with less affinity	Lower affinity for glucose compared to mannose and fructose, high capacity
SGLT 6	Intestine, kidney, brain	Transport D-chiro-inositol and myo-inositol with high affinity	Low affinity for glucose, capacity unknown

Adapted from Navale and Paranjape (1).

**Figure 2:**
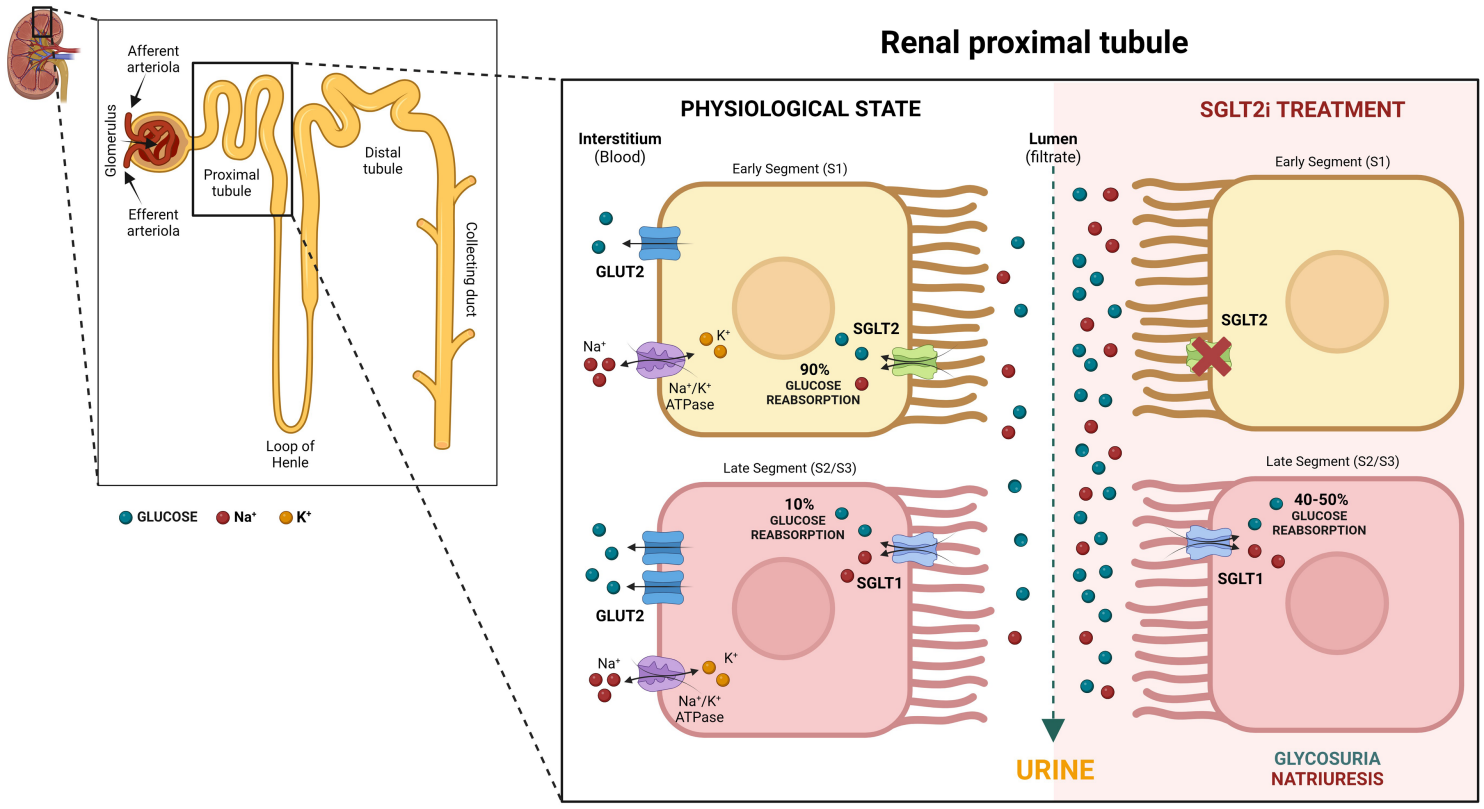
Role of SGLT2 and SGLT1 in glucose re-absorption within the proximal renal tubules with and without SGLT2 inhibition. Created in BioRender. Uerj, L. (2025) https://BioRender.com/v55m360.

In the kidney, glucose is freely filtered from the blood at the glomerulus. To avoid losing significant amounts of the main body’s fuel into the urine, glucose reabsorption occurs in the renal tubules. Both SGLTs type 1 and 2 are proteins in the luminal border of the epithelial cells of the proximal convoluted tubules, reclaiming glucose from the kidney filtrate and returning it to the blood (Figure 2). Filtered glucose first encounters SGLT2 in the initial segment of the proximal renal tubules, where it co-transports one sodium ion with one molecule of glucose across the membrane (1, 6). Previously thought to be located in the early S1/S2 segment, new refined techniques have suggested that SGLT2 is solely located in the S1 segment of the proximal renal tubules, at least in rats (7). The SGLT2 is a high-capacity, low-affinity glucose transporter responsible for 90 percent of the total glucose absorption in the kidney (Figure 2). The SGLT1 is found in a later segment of the proximal tubule (S2 and S3), transporting one molecule of glucose or galactose with two sodium ions across the membrane (1, 6). SGLT1 is a lower-capacity, high-affinity transporter that accounts for 10% of glucose reabsorption (Figure 2). Because of their role in glucose reabsorption, SGLTs became a drug target for glycemic control.

This narrative review focuses on a recent Food and Drug Administration (FDA) approved class of hypoglycemic drugs known as SGLT2 inhibitors (SGLT2i) to treat diabetes mellitus (DM) in cats. We summarize the human data that supports the use of SGLT2i in controlling human type-2 DM and protecting against the worsening of certain DM comorbidities. We also review the available literature regarding the effects of SGLT2i on cats.

## 2 Search strategy

One author (ABV) searched PubMed and Google Scholar for “SGLT inhibitors and diabetes mellitus,” “SGLT inhibitors and heart,” “SGLT inhibitors and kidney,” and “SGLT inhibitors and cognitive impairment.” Several combinations including the terms, “humans,” “cats,” “veterinary,” “SGLT1,” “SGLT2,” “review,” “cardioprotection,” “renal protection,” and “neuroprotection” were used to identify relevant publications up to June 2024. Manual scoping of results focused on original research articles, meta-analyses, systematic reviews, and narrative reviews. The eligibility criteria included: all relevant literature on SGLTi in humans and cats, relevant supporting literature regarding the physiology of SGLTs and pharmacology of SGLT2i, peer-reviewed articles written in English, and full-text articles.

## 3 SGLTs as pharmacological targets

The first known substance with SGLT inhibitory activity, Phlorizin, is a glycoside phytocompound isolated from the root bark of an apple tree in 1835 (8). Phlorizin showed high affinity, specificity, and competitive inhibition capacity for SGLT1 and SGLT2 (9, 10). Since then, several phlorizin analogs (gliflozins) with different potency and selectivity against SGLT1 and SGLT2 have been developed and approved for human use worldwide.

Canagliflozin was the first SGLT2i approved in the United States (March 2013) for use in human adults with type 2 diabetes. Canagliflozin has 400-fold higher inhibitory activity for SGLT2 over SGLT1 (11). Currently, at least six high-potency gliflozins are approved for use in humans in the United States (Table 2). Recently, the FDA approved two gliflozins for the treatment of diabetes mellitus in cats: velagliflozin in December 2022 (Bexacat®; Elanco) and velagliflozin (12) in July 2023 (Senvelgo®; Boehringer Ingelheim) both with high affinity for SGLT2 inhibition. FDA-approved gliflozins for humans and cats are summarized in Table 2.

**Table 2:** Approved high-potency sodium-glucose transporter 2 inhibitors (SGLT2i) for use in humans and cats in the United States.

Generic agent	Brand	Year FDA-approved	Target population	FDA-approved indications for all SGLT2i	Off-label uses for all SGLT2i
Canagliflozin	Invokana	2013	Human adults^*^	Improvement of glycemic control in type 2 DMType 2 DM and established CVS diseaseDecrease the risk of CVS hospitalization and death for HF in HFrEF patientsDecrease the risk of eGFR decline and hospitalization in CKD patientsImprovement of CVS outcome in HFpEF patientsApproved for HF treatment across the full spectrum of LVEF patients	Management of obesity in combination with GLP-1 receptor agonistsNonalcoholic fatty liver diseaseAlzheimer’s disease
Dapagliflozin	Farxiga	2014	Human adults^*^		
Empagliflozin	Jardiance	2014	Human adults^*^		
Ertugliflozin	Steglatro	2019	Human adults^*^		
Bexagliflozin	Brenzavvy	2023	Human adults^*^		
Sotagliflozin	Infepa	2023	Human adults^*^		
Bexagliflozin	Bexacat	2022	Cats	Diabetes Mellitus	Insulin resistance in obesity^a^Enhancement of GFR^b^
Velagliflozin	Senvelgo	2023	Cats		

FDA, Food and Drug Agency; DM, Diabetes Mellitus; CVS, cardiovascular; HF, heart failure; HFrEF, HF with reduction ejection fraction; GFR, glomerular filtration rate; CKD, chronic kidney disease; HFpEF, HF with preserved ejection fraction; LVEF, left ventricular ejection fraction; GLP-1, glucagon-like peptide-1.

* FDA has not yet approved SGLT2is for use in children.

a Hoenig et al. (118).

b Gal et al. (130).

The initial rationale behind gliflozins’ development was to manage hyperglycemia by inhibiting the glucose uptake from the proximal renal tubule, thereby allowing the kidneys to dispose of excess blood glucose in the urine (Figure 1). However, after years of clinical studies, there is strong evidence that SGLT2 inhibitors are more than hypoglycemic agents and act as pleiotropic drugs with significant metabolic, cardiovascular, renal, and possibly neuroprotective benefits in humans (13–18).

## 4 SGLT2i treatment in humans with DM and possible mechanism of action

Gliflozins act to improve diabetic hyperglycemia by inhibiting SGLT2, which is responsible for 90% of the glucose filtered at the glomerulus (19). However, physiological changes that occur in response to SGLT2i increase the resorptive capacity of SGLT1 and maintain filtered glucose reabsorption at around 50% (20) (Figure 2). This compensatory reabsorption may reduce the risk of hypoglycemia, which is a rare event in human patients using SGLT2i (21).

**Figure 3:**
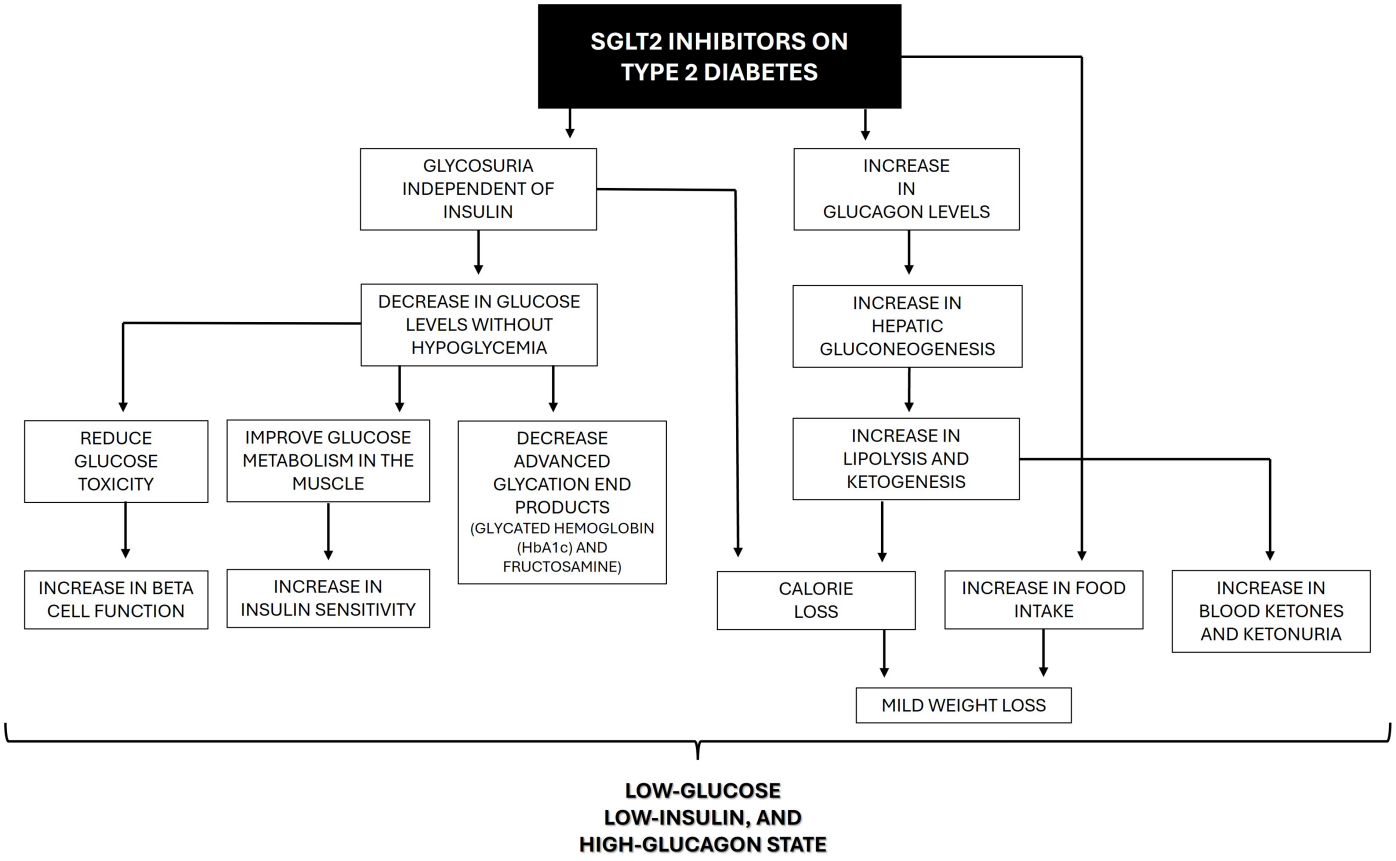
Beneficial effects of SGLT2 inhibitors on the pathophysiology of type 2 diabetes in humans.

Soon after the inhibition of SGLT2, glycosuria will appear, and consequently, blood glucose concentration will decrease independent of insulin. An improvement in beta cell function is expected because of the reduction of glucotoxicity (22). Moreover, improvements in skeletal muscle insulin sensitivity, positively correlated with the improvement in daily plasma glucose fluctuations, were reported, possibly secondary to the improvement in mitochondrial oxidative phosphorylation (23). Interestingly, the glucose-lowering effect of SGLT2i in humans is considered modest and probably insufficient to account for all clinical benefits demonstrated in patients using these drugs so far (15). The fall in glucose levels is less than might be expected because glucagon levels increase, contributing to an increase in hepatic glucose production (22). Chronic treatment with SGLT2i also increases lipolysis and a shift to fat oxidation, which results in weight loss. Nonetheless, this effect is presumably counteracted by the compensatory increase in food intake, and the weight loss is usually considered mild. Additionally, with time, glucose oxidation decreases, and lipid oxidation increases without affecting protein oxidation. These changes occur probably because of the low-glucose, low-insulin, and high-glucagon state and maintain energy balance in the long term (Figure 3) (22).

**Figure 4:**
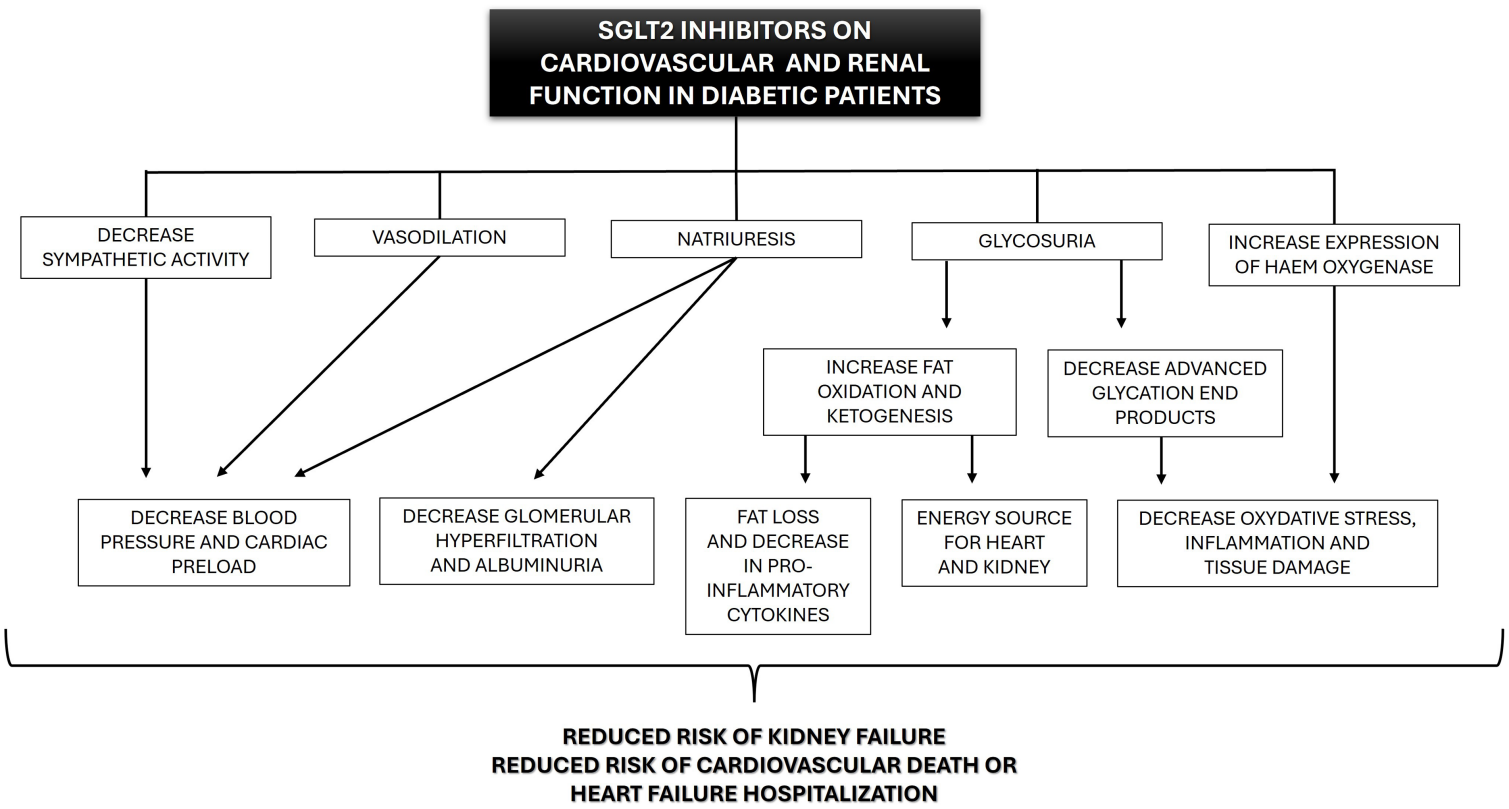
Beneficial effects of SGLT2 inhibitors (SGLT2i) on cardiovascular and renal function in diabetic human patients.

Many of the benefits that are now recognized were not anticipated when SGLT2i was initially developed. Through different mechanisms, some still unknown, gliflozins produce a cascade of physiological benefits in major organ systems usually affected by DM (Figure 4).

In addition to the hypoglycemic effect, gliflozins act as proximal and osmotic diuretics, inducing natriuresis (21). This effect comes from the reduced activity of Na^+^/H^+^ exchanger 3 (NHE3), which, in normal conditions, reabsorbs 30% of the sodium in the proximal tubule. NHE3 is closely regulated by glucose metabolism and SGLT transporters, which makes it sensitive to SGLT2i (24). In diabetic patients, NHE3 activity is enhanced (hyper-reabsorption) and contributes to the decreased sodium availability at the macula densa, afferent arteriole vasodilation, increased intraglomerular pressure, and associated hyperfiltration (21). This glomerular hyperfiltration eventually leads to fibrosis within the glomerulus and tubulointerstitium and to proteinuria (25, 26). Under the action of SGLT2i, the unabsorbed sodium will be delivered to the macula densa cells in the distal nephron, normalizing afferent arteriole tone, intraglomerular pressure, and glomerular filtration, which probably decreases protein filtration and tubulointerstitial damage (21, 27, 28). In addition, previous research showed that natriuresis is likely responsible for the decrease in total sodium retention (21), tissue sodium content (29), and the reduced interstitial volume relative to blood volume (30). These effects may ameliorate fluid congestion with less impact on tissue perfusion compared to other diuretics (15). Furthermore, SGLT2i have electrolyte-sparing advantages compared to classical diuretics (thiazides and loop diuretics). Although the pathophysiological mechanisms remain poorly understood, in general, potassium, magnesium and sodium levels seem to be unaffected by SGLT2i treatment (31–34). These properties may provide greater flexibility regarding dose optimization for renin-angiotensin-aldosterone system inhibition drugs and mineralocorticoid receptor antagonists, which can be limited by hyperkalemia, particularly with kidney function decline (35).

Gliflozins also seem to have antioxidant and anti-inflammatory effects. Antioxidant effects were related to the increased expression and activity of haem oxygenase, an enzyme involved not only in the degradation of haem but also in reducing oxidative stress, inflammation, apoptosis, and thrombosis (36–39). The anti-inflammatory effects of SGLT2i might be the result of combined actions. First, SGLT2i reduces the production of pro-inflammatory cytokines, including IL-6 and IL-8 (40–42). Second, these drugs increase β-oxidation of free fatty acids, increasing ketone production, especially in diabetic patients (43–45). Last, the ketone β-hydroxybutyrate can suppress pro-inflammatory cytokine release (e.g., IL1β and IL-18) by blocking the murine and human NLPR3 (NLR family pyrin domain containing 3), a protein expressed predominantly in macrophages and as a component of the inflammasome. Activated NLRP3 would trigger an immune response (46). Furthermore, patients using canagliflozin for over 2 years showed reduced levels of markers and mediators of fibrosis compared to patients receiving glimepiride (47). It is unclear if this anti-fibrotic action is mediated by antioxidant and anti-inflammatory mechanisms or independent anti-fibrotic mechanisms (48).

In addition to the above mechanisms, SGLT2i may reduce sympathetic hyperactivity (49–51), which is a common finding in patients with diabetes, obesity, hypertension, and chronic kidney disease, and can lead to vasoconstriction and increased risk of fatal arrhythmias (52, 53).

Finally, treatment with gliflozins also seems to induce vasodilation and reduce vascular resistance in animal models, probably secondary to an increase in the bioavailability of nitric oxide (54, 55). The effects induced by nitric oxide likely add to the vascular benefits of sympathetic inhibition and together could contribute to the favorable cardiac reverse remodeling (e.g., reduction in ventricular mass) reported with SGLT2i use (56).

## 5 Cardiovascular protective effects of SGLT2i in humans

Atherosclerotic cardiovascular disease (CVD) is the leading cause of morbidity and mortality in people with DM. Currently, the FDA recommends cardiovascular (CV) outcomes trials (CVOTs) for all new hypoglycemic drugs to monitor the safety of these agents in people with either established CVD or at higher risk of development of CVD. Furthermore, clinical guidelines are frequently based on CVOTs results (57, 58). Some of the most convincing data about CV protective effects of SGLT2i came from early CVOTs using canagliflozin and empagliflozin (59, 60). These data showed that SGLT2i could reduce the risk of cardiovascular events (e.g., hospitalization for heart failure and cardiovascular death) in diabetic patients with established atherosclerotic cardiovascular disease (60). Subsequent trials in patients with heart failure and reduced or preserved left ventricular ejection fraction demonstrated that SGLT2i also has beneficial effects on heart failure outcomes (15). At present, both the American College of Cardiology (ACC) and the European Society of Cardiology (ESC) 2024 updates recommend the use of SGLT2i (dapagliflozin and empagliflozin) for patients with heart failure with or without diabetes mellitus (57, 58).

## 6 Renal protective effects of SGLT2i in humans

Diabetic nephropathy is a major complication of DM in humans, which results in chronic kidney disease (CKD) (61, 62). Humans with DM are 10 times more likely to develop end-stage kidney failure, and 40% of diabetic patients might develop the final stage of this disease (63). The nephroprotective effects of SGLT2 inhibitors in humans are well established and have been tested in randomized controlled trials in nearly 100,000 human adults (64). Research trials using empa-, cana- and dapagliflozin showed impressive results in renal outcomes for patients with DM (59, 60, 65). SGLT2i reduces the risk of kidney disease progression in people with or without diabetes and the risk of acute kidney injury. Furthermore, since SGLT2i do not affect serum potassium levels, there is a reduced risk of hyperkalemia compared to other drugs commonly used in diabetic nephropathy (e.g., angiotensin-converting enzyme inhibitors) (18). Because SGLT2i significantly reduces or normalizes the glomerular hyperfiltration seen in diabetic patients, there is probably a decrease in the physical stress placed on glomerular capillaries. By decreasing the glomerular filtration of tubulo-toxic factors (e.g., albumin and advanced glycation end products), there is a decrease in hypoxia, oxidative stress, inflammation, fibrosis, and progression of CKD (66). Currently, empagliflozin and dapagliflozin are approved for the treatment of CKD with or without diabetes mellitus in human adults. No research or approval exists for the use of gliflozins in children with CKD (64).

## 7 Other benefits of SGLT2i treatment (blood pressure, weight loss, hepatic lipidosis, neuroprotection)

The benefits of SGLT2 in humans go beyond the hypoglycemic effect and cardiovascular and renal protection. Gliflozins can modestly decrease systolic and diastolic blood pressure without significantly increasing the risk of hypotensive episodes (67). Additionally, the glycosuric effect of SGLT2i results in calorie loss (elimination of 60–80 g of glucose per day in the urine), which consequently induces mild weight loss from the first weeks of treatment, which could be maintained for up to 4 years (19, 68–75). Furthermore, treatment with SGLT2 improves liver outcomes in people with metabolic dysfunction-associated steatotic liver disease (43, 76–82). Moreover, since SGLT2i still have some affinity for SGLT1 receptors in the brain and can cross the blood–brain barrier, they have been studied for neuroprotective effects (83–85). Gliflozins demonstrate anti-inflammatory and antioxidant properties in the nervous system and inhibit acetylcholinesterase, which could contribute to cognitive improvement (16, 86).

## 8 Ketoacidosis, urogenital infection, and SGLT2i treatment in humans

Placebo-controlled trials and real-world cohort studies using gliflozins have reported favorable adverse effect profiles of these drugs in humans (15, 17, 87). Nevertheless, there is up to 0.3% increase in the risk of ketoacidosis development in people with diabetes using SGLT2i (18, 88). Although ketoacidosis is usually associated with hyperglycemia, glucose levels can be normal or modestly elevated in affected patients treated with SGLT2i, so-called euglycemic ketoacidosis (89, 90). The mechanisms by which gliflozins slightly increase the risk of DKA are probably related to reduced insulin secretion or increased insulin resistance and stimulation of glucagon secretion, increasing ketone body synthesis (91). In human medicine, this increased risk is mitigated by using several strategies, including withholding the agent when unwell and stopping therapy 3 days before any procedure that requires fasting, bowel preparation, or hospital admission (90).

No increased risk of urinary tract infection has been reported in SGLT2 trials (92). On the other hand, humans with DM receiving therapy with SGLT2 inhibitors were associated with 2.3–6.4% increased risk of genital mycotic infections in major clinical trials (93–95). Studies reporting the same risk in people without DM who received SGLT2i have had conflicting results (96–99).

## 9 Current knowledge about SGLT2i treatment in cats with DM

Until recently, diabetic cats were primarily treated with insulin injections and a high-protein, low-carbohydrate diet (100, 101). Although insulin is an effective treatment, it requires daily injections and personal commitments, which can impact the daily routine and quality of life of owners and cats (102–105). Several oral hypoglycemic drugs have been evaluated in healthy, experimentally hyperglycemic, obese, and diabetic cats in the past (106–111). Overall, only sulfonylurea glipizide is an acceptable treatment option in some cats (112, 113). Because of its mechanism of action stimulating insulin and amylin production, sulfonylureas can increase pancreatic amyloidosis and lead to beta-cell destruction (114–116). While both insulin and glipizide can induce hypoglycemia in cats, SGLT2i demonstrate an overall lower risk of hypoglycemia in humans when compared to other drugs (117).

In the last few years, studies have emerged highlighting the utility of SGLT2i in the treatment of feline DM. One study evaluated for the first time the effect of velagliflozin in obese cats (118). Placebo or velagliflozin (1 m/kg, PO, q24 h) was administered to two groups of six neutered obese cats matched by gender for 35 days. Authors documented an increase in urinary glucose excretion and suggested that velagliflozin could be beneficial for the treatment of diabetic cats (118). Using 252 newly diagnosed and insulin-treated cats, another study evaluated the effect of velagliflozin once daily as a standing-alone therapy compared to insulin injection therapy (119). Cats were administered velagliflozin (1 mg/kg, PO, q24 h) regardless of blood glucose level, and evaluated on days 2 or 3, and days 7 and 30 and then monthly. Of the 252 cats enrolled, only 198 were evaluated. From this population, 175 (88.4%) were considered a treatment success on day 30 based on improved glycemic control and clinical signs. The most common adverse effects were diarrhea and vomiting, and the most serious adverse event was DKA, which occurred in 5% of the naïve diabetic cats and 18% of cats previously treated with insulin (119).

Recently, a prospective, randomized, positive controlled, open-label, noninferiority field trial using client-owned diabetic cats (127 safety and 116 efficacy assessment) was published (120). Authors compared velagliflozin (1 mg/kg PO, q24 h) with porcine lent insulin (titrated Caninsulin, q12 h) and concluded that velagliflozin treatment was non-inferior to Caninsulin injections, and cats showed good quality of life and glycemic control without developing clinical hypoglycemia (120). Although porcine lent insulin can be used for cats, the author’s choice of insulin in cats is studies comparing SGLT2i and basal insulins like glargine will.

A second SGLT2i, called bexagliflozin, induced maximal renal glucose excretion at a dose of 15 mg/cat, PO, q24 h during pre-clinical research, according to the manufacturer (121). Not many studies have investigated this drug in cats so far (122, 123). One study evaluated the effect of bexagliflozin associated with insulin in five client-owned cats with poorly controlled DM (122). Cats were treated with bexagliflozin for 4 weeks, and all of them had a significant reduction in insulin dose requirement, and insulin was discontinued in two cats. Moreover, there was a significant decrease in blood glucose concentration obtained from blood glucose curves. No cats had any documented hypoglycemic episode, and adverse effects were considered mild (122). Another clinical trial evaluated the safety and effectiveness of bexagliflozin (15 mg/cat, PO, q24 h) as a monotherapy for newly diagnosed diabetic cats (123). In an open-label, historically controlled prospective clinical trial, authors evaluated the effect of bexagliflozin (15 mg/cat, PO, q24 h) in client-owned cats. Of the 84 cats enrolled, only 81 were evaluated on day 56. Eighty-four percent of these cats were considered treatment successes based on improvements in glycemic control and clinical signs. Commonly observed adverse events included emesis, diarrhea, anorexia, lethargy, and dehydration. The most important adverse event recorded was euglycemic DKA, diagnosed in three cats (3.6%) and presumed present in a fourth (123).

While the results of SGLT2i use in cats are promising, studies have not been conducted as independent clinical trials. Additionally, long-term oral medication can be challenging for some cats, even if it is taken once daily, and SGLT2i treatment could require the same life-long commitment as insulin usually does. Furthermore, bexagliflozin and velagliflozin are unavailable worldwide and may be cost-prohibitive for some cat owners. The dose recommendation is once daily, but not all diabetic cats are candidates for this monotherapy. The suggested criteria for use in newly diabetic cats can be found elsewhere (124, 125). When this review was published at the beginning of 2025, veterinary bexagliflozin was priced at around $ 120, and velagliflozin at around $ 280 per month per cat in the USA. For comparison, a bottle of human bexagliflozin (Brenzavvy®, 20 mg/30 tablets) is currently priced around $50, and a bottle of 10 mL of porcine lent insulin (Caninsulin®/Vetsulin®, Merck) costs around $70, which, according to the manufacturer, should be discarded after 42 days. Also, a 3 mL pen of glargine insulin costs around $100 and, in the author’s (ABV) experience, can be used safely for up to 3 months if stored in the fridge, considering that most cats receive 1–3 U/BID/daily.

Studies in diabetic cats using human SGLT2i, like dapagliflozin, are needed. This drug, for example, is one of the most popular in human medicine. The same brand (Farxiga® 10 mg/30 tablets) is widely available at lower costs in countries like Brazil ($38), Australia ($43), Canada ($76), and the United Kingdom ($90). Finally, SGLT2i treatment is compatible with most other glucose-lowering agents (126), and used in human patients with a wide range of comorbidities, but currently, its use in diabetic cats with concurrent illnesses is discouraged (124, 125).

## 10 Future perspectives for the use of SGLT2i in cats with other diseases

As in humans, cats commonly develop CV disease, CKD, obesity, hepatic lipidosis, and cognitive impairment as they age. Most of these diseases share similarities with human conditions, and some, like DM and cognitive dysfunction syndrome, were already considered a natural animal model for human studies (127–129). Currently, there is very limited knowledge regarding the effects of SGLT2i on other feline diseases.

A study evaluated the effect of velagliflozin (1 mg/kg/PO, q 24 h, for 35 days) or placebo in two groups of six neutered adult obese cats (118). Different parameters were evaluated before and after treatment. Significant changes after treatment with velagliflozin included a decrease in respiratory exchange ratio, an increase in cholesterol, a small increase in albumin, and a rise in beta-hydroxybutyrate and nonesterified fatty acids. Less insulin was secreted during an intravenous glucose tolerance test, suggesting improved insulin sensitivity. Treatment did not affect the intravenous insulin tolerance test, glucagon, leptin, or adiponectin. Water intake, urine output, urinary glucose excretion, and the glucose/creatinine ratio but not urinary electrolytes were significantly higher post-treatment (118). Currently, there are no studies regarding the long-term effects of SGLT2is in obese cats with or without DM.

A randomized 2-way controlled crossover study investigated the effect of dapagliflozin on glomerular filtration rate in eight adult castrated male healthy cats (130). Cats received or not 10 mg of SGLT2i per day for 5 days in each of the four trial periods, with washout periods of 7 days in between. Urine and blood were sampled on the first and fifth day of each trial to analyze serum urea, creatinine, symmetric dimethylarginine, and 24-h sodium and chloride urinary excretion. Glomerular filtration rate was accessed using iohexol clearance on the fifth day of each trial. Compared to controls, healthy cats treated with dapagliflozin significantly increased mean glomerular filtration rate. No significant changes were seen for other parameters. Authors believed that dapagliflozin-mediated delivery of sodium and glucose distal from the proximal convoluted tubule induced compensatory increased sodium absorption at the thick ascending loop of Henle that resulted in decreased sodium delivery to the distal tubule leading to tubuloglomerular feedback-mediated glomerular hyperfiltration (130). Currently, there are no studies on the effects of SGLT2i in cats with CKD or naturally occurring heart disease.

## 11 Conclusion

SGLT2 inhibitors have opened new possibilities for managing type-2 DM in both humans and cats. Cats share many similarities with human diseases and can develop obesity, heart failure, CKD, hepatic lipidosis, and cognitive dysfunction as they age. Future research in small animal medicine must address the benefits and adverse effects of SGLT2i treatment not only as a new hypoglycemic agent for feline DM but also as a pleiotropic drug with expected effects in many other physiological systems.
